# A Minimal-Invasive Metabolic Test Detects Malignant Hyperthermia Susceptibility in a Patient after Sevoflurane-Induced Metabolic Crisis

**DOI:** 10.1155/2013/953859

**Published:** 2013-12-26

**Authors:** Frank Schuster, Stephan Johannsen, Norbert Roewer

**Affiliations:** Department of Anesthesia and Critical Care, University of Wuerzburg, Oberduerrbacher Street 6, 97080 Wuerzburg, Germany

## Abstract

Malignant hyperthermia is a rare but life-threatening complication of general anesthesia in predisposed patients usually triggered by potent inhalation anesthetics and/or the depolarizing muscle relaxant succinylcholine. The authors present a case of delayed sevoflurane-induced malignant hyperthermia in a 21-year-old male patient that was sufficiently treated by discontinuation of trigger agent application and dantrolene infusion. After surviving an MH episode diagnostic procedures are indicated to increase patient safety. In the presented case, the use of a novel minimal-invasive metabolic test with intramuscular injection of halothane and caffeine successfully confirmed MH susceptibility and hence might be an alternative for invasive in vitro contracture testing in selected cases.

## 1. Introduction

Malignant hyperthermia (MH) is a rare but potentially lethal pharmacological induced disease of skeletal muscle. Exposure to triggering agents such as volatile anesthetics and/or the depolarizing muscle relaxant succinylcholine may induce a hypermetabolic muscular syndrome characterized by hypoxemia, hypercapnia, tachycardia, muscular rigidity, acidosis, hyperkalemia, and hyperthermia, due to an uncontrolled sarcoplasmic calcium release via functionally altered ryanodine receptors subtype 1 or dihydropyridine receptors [1]. Currently, the in vitro contracture test (IVCT) requiring an open muscle biopsy is the only reliable procedure to diagnose MH susceptibility in affected patients. However, due to its invasive characteristics this test is associated with severe risks to the patients, for example, wound infections, postoperative bleeding, or persistent dysesthesia. Important progress was made within the last years by screening for causative MH mutations, which allows a genetic diagnosis in 30% to 50% of MH families [2]. Unfortunately, a negative genetic result does not sufficiently exclude MH susceptibility and hence must be confirmed by IVCT [3]. Recently, a minimal-invasive metabolic test was proposed to analyze muscular alterations in MH patients under in vivo conditions. The local monitoring of interstitial lactate concentrations after pharmacological stimulation induced by MH trigger agents allowed a differentiation between MH susceptible (MHS) and MH nonsusceptible (MHN) patients [4]. 

In the presented case report, we used this minimal-invasive test to screen for MH susceptibility in a patient who developed clinical sings of MH during general anesthesia with sevoflurane while undergoing an elective shoulder arthroscopy. 

## 2. Case Presentation

### 2.1. Intra- and Postoperative Course

With approval of the local ethics committee (application number: 263/11, ethics committee of the University of Wuerzburg), we report the case of a 21-year-old male patient weighting 100 kg, who was scheduled for elective shoulder arthroscopy. Neither the patient nor his family had any history of neuromuscular disease or MH. According to the medical records, the patient underwent two uneventful anesthesias using halothane combined with oxygen (O_2_) and nitrous oxide (N_2_O) for cleft lip revision at the age of 5 months and at the age of 4 years, respectively. The preoperative laboratory examinations were within normal values. Initial heart rate (95 bpm), blood pressure (145/80 mmHg), and peripheral oxygen saturation (96%) were unremarkable. Anesthesia was induced by intravenous application of 0.1 mg/kg piritramid, an initial bolus of 2.5 mg/kg propofol followed by an additional application of 1.5 mg/kg propofol, and 1.5 mg/kg succinylcholine. To secure patient's airways, a size 8,0 mm cuffed endotracheal tube was inserted after direct laryngoscopy. Afterwards, anesthesia was maintained by sevoflurane 1.5 vol% supplemented by O_2_/N_2_O and application of 0.05 mg/kg piritramid if needed. During the initial period of surgery, hemodynamic and metabolic parameters were within normal limits with end-tidal carbon dioxide values between 38 and 39 mmHg. Suddenly, after 290 min a slightly increase of heart rate from 60 bpm to 80 bpm and a rise in systolic blood pressure from 120 mmHg to 135 mmHg were noticed. Furthermore, end-tidal carbon dioxide concentration rapidly increased from 39 mmHg to 85 mmHg within 5 min after the onset of sinus tachycardia. Simultaneously, oxygen saturation decreased from 98% to 93%. Unfortunately, there was no monitoring of body temperature, but the attending anesthesiologist observed and documented warming of head and chest during this episode. After MH was suspected, the anesthesiologist immediately stopped sevoflurane and hyperventilated the patient with 100% oxygen (25 L/min). In addition, 240 mg dantrolene was applied twice and anesthesia was continued intravenously by infusion of 5 mg/kg/h propofol and repeated fentanyl applications. After these interventions, hemodynamic and metabolic parameters were stabilized within 10 min. The surgical procedure was stopped, and the patient was transferred to the intensive care unit (ICU) in stable conditions. 

Laboratory analyses performed after admission to the ICU detected a significant rhabdomyolysis with creatine kinase levels about 20.000 U/L and a hyperkalemia (6.4 mmol/L). Further laboratory data were unremarkable. Interestingly, blood gas analysis drawn one hour after the MH suspected event did not show signs of metabolic or respiratory acidosis. Four hours after the admission, the patient was extubated without neurological deficits. 

## 3. Diagnostic Findings

Due to the suspected MH event, the patient was informed about the possible risk of MH susceptibility and a diagnostic workup was recommended. Hence, after written and oral informed consent of the patient, we decided to perform the recently developed minimal-invasive metabolic test three days after the suspected MH episode. In brief, after regional anesthesia of the skin, two microdialysis probes with a semipermeable membrane for measurement of interstitial metabolites were inserted into the lateral vastus muscle and perfused with 1 *μ*L/min Ringer's solution. 15 min after equilibration either a single bolus of 200 *μ*L halothane 4 vol% dissolved in soy bean oil or 200 *μ*L caffeine 80 mM was injected into the muscular tissue. Dialysate samples were collected after 15-minute intervals, and lactate concentration was measured spectrophotometrically. If lactate values exceeded a threshold of 2.8 mM after halothane or 1.6 mM after caffeine, which had been defined by a previous set of tests, MH susceptibility was assumed [4]. Prior to halothane or caffeine application, the baseline lactate levels did not significantly differ between both microdialysis probes (0.4 mM versus 0.8 mM). After halothane injection the lactate values significantly increased to a maximum of 3.7 mM. Similarly, caffeine induced a significant increase of lactate to 3.1 mM (Table 1).

Even though MH susceptibility had been confirmed by this metabolic test, the patient decided to undergo further diagnostic testing due to personal reasons. Due to the MH suspected course of the described case and to avoid invasive IVCT, we determined to screen the hotspots of the ryanodine receptor subtype 1 gene for MH related alterations. Unfortunately, genetic analysis did not detect MH associated mutations. Hence, twelve weeks after the MH suspected event an open muscle biopsy and IVCT according to published guidelines of the European MH Group were performed at our lab [3]. In few words, 2.5 g muscle tissue was excised of the left vastus lateral muscle after femoral nerve block. Single muscle bundles were mounted in a tissue bath and exposed to incremental concentrations of caffeine (0.5; 1; 1.5; 2; 3; 4; and 32 mM) or halothane (0.11; 0.22; 0.44; and 0.66 mM) at 3 min intervals. Since significant contractures ≥ 2 mN occurred at the defined threshold concentrations of caffeine 2 mM and halothane 0.44 mM, the MH-susceptibility of the patient was confirmed (Table 2).

## 4. Discussion

Malignant hyperthermia is a rare but life-threatening complication of general anesthesia usually triggered by volatile anesthetics and/or succinylcholine in susceptible patients. While genetic frequency of MH predisposition is stated to be 1 : 2.000, the prevalence of MH-episodes varies regionally between 1 : 10.000 and 1 : 220.000 [5]. 

Nowadays, due to continuous progress in anesthesia, it seems that the incidence of fulminant MH crisis decreases. Since the potent MH trigger halothane is no longer used in clinical routine in industrialized countries, the presently utilized inhalation anesthetics appear to delay the onset of MH or lead to abortive MH reactions with alleviated symptoms. For instance, Hopkins and colleagues reported that the onset of MH was significantly faster after halothane exposure (median: 20 min, range: 5–45 min) compared to sevoflurane (median: 60 min, range: 10–210 min) [6]. Hence, the intraoperative course in the presented case with development of MH signs 290 min after induction of general anesthesia seems consistent with these findings. However, in some cases application of sevoflurane induces MH symptoms within few minutes [7]. 

In addition, anesthesiologists must be aware that previous uneventful anesthesia does not exclude MH susceptibility [8]. On average, susceptible patients undergo three uneventful anesthesias until the first MH episode occurs [5]. In this context, it is not surprising that the presented patient reported two unremarkable anesthesias in the past. The underlying pathomechanism why some patients develop MH during the first exposition to triggering agents while others do not still remains unclear. A possible explanation might be the presence of an individual compensation mechanism at a cellular level lowering myoplasmic calcium concentrations in these patients.

Based on these observations, attending anesthesiologists must keep in mind that MH may occur at any time during general anesthesia and prior unremarkable anesthesias, are not a prove for the absence of MH susceptibility. 

In case of a suspected MH, application of trigger agents must be discontinued immediately and causal therapy by dantrolene infusion should be initiated to avoid serious harm to the patient [9]. The mode of action of dantrolene is based on inhibition of the sarcoplasmic calcium release during an MH episode without increasing sarcoplasmic calcium reuptake [10]. The return of metabolic parameters to normal values reflects the therapeutic success of MH treatment, comparable to the absent of metabolic or respiratory acidosis in the presented case. 

Due to the possible risk in case of future anesthesia, patients should be referred to a MH-center to initiate further diagnostics after surviving a suspected MH event. In the reported case, the authors decided to apply a novel minimal-invasive metabolic test with intramuscular halothane and caffeine injection to screen for MH susceptibility. Based on the metabolic alterations in the course of an MH episode, measurement of interstitial lactate concentration was assumed as a suitable method to detect MH in affected patients. The measured increase of local lactate concentrations following halothane and caffeine application clearly indicated the diagnosis “MHS” in our patient. In contrast to the IVCT, the metabolic test is less invasive; since the induced metabolic reactions are limited to an area < 10 mm around the inserted microdialysis probes and due to the expected dilutional effects of the administered drugs in the tissue, serious systemic, or local adverse effects are unlikely [11]. Furthermore, previous histological examination of rat muscular tissue after application of caffeine 80 mM revealed only unspecific morphological changes [12]. 

Although MH susceptibility was proven by the metabolic test, the patient decided to undergo further diagnostic testing. Due to the clinical course of the reported MH reaction and in contrast to the diagnostic guidelines of the European MH Group, we decided to perform genetic screening at first. Unfortunately, no mutation of the ryanodine receptor subtype 1 could be detected in the patient. Hence, IVCT was carried out eight weeks after the suspicious event, since the absence of a mutation does not reliably exclude MH [13]. 

In summary, the authors present a case of a delayed sevoflurane-induced MH in a 21-year-old male patient, who was sufficiently treated by dantrolene and discontinuation of trigger agent application. After surviving an MH episode diagnostic procedures are indicted to increase patient safety. In the presented case, the use of a novel minimal-invasive metabolic test with intramuscular application of MH triggering agents such as halothane and caffeine successfully proved MH susceptibility and hence might avoid invasive in vitro contracture testing in selected cases.

## Figures and Tables

**Table 1:** Local lactate increase following interstitial application of 200 *µ*L caffeine 80 mM and 200 *µ*L halothane 4 vol%.

Time	0 min	15 min	30 min	45 min	60 min	75 min
Caffeine 80 mM	0.4 mM	0.6 mM	0.7 mM	2.7 mM	3.1 mM	2.7 mM
Halothane 4 vol%	0.8 mM	0.7 mM	0.9 mM	2.1 mM	3.7 mM	2.8 mM

0 min: baseline lactate levels; 15 min: time point of caffeine or halothane injection.

**Table 2:** In vitro contracture test results. A contracture ≥ 2 mN at the defined threshold concentrations of caffeine 2 mM and halothane 0.44 mM confirmed MH susceptibility.

Caffeine	Predrug	0,5 mM	1 mM	1,5 mM	2 mM	3 mM	4 mM	32 mM
Test 1	14.0 mN	13.5 mN	15.6 mN	19.6 mN	25.6 mN	29.1 mN	29.1 mN	144 mN
Test 2	12.7 mN	12.2 mN	13.0 mN	17.8 mN	24.7 mN	35.7 mN	32.2 mN	209 mN

Halothane	Predrug	0,11 mM	0,22 mM	0,44 mM	0,66 mM			

Test 1	11.6 mN	23.0 mN	35.1 mN	32.1 mN	26.5 mN			
Test 2	19.1 mN	20.9 mN	28.1 mN	29.5 mN	26.8 mN			
